# The Effect of an Education-Based Intervention on Self-Reported Awareness and Practice of Iranian Nurses in Observing Patients’ Rights

**DOI:** 10.5539/gjhs.v7n3p98

**Published:** 2014-11-17

**Authors:** Kobra Abedian, Masumeh Bagheri Nesami, Zohreh Shahhosseini

**Affiliations:** 1 Department of Reproductive Health, Nasibeh Nursing and Midwifery Faculty, Mazandaran University of Medical Sciences, Sari, Iran

**Keywords:** nurses, patient’s rights, human right, Iran

## Abstract

**Background:**

For patients’ rights to be observed, first patients and health care providers should be aware of these rights. Nurses’ lack of awareness of these rights leads to their inability to recognize patients’ legal and ethical issues, and reduces the quality of provided services. This study was conducted to determine the effect of an education-based intervention on self-reported awareness and practice of nurses in observing patients’ rights.

**Methods:**

In this quasi-experimental study, awareness and practice of 90 nurses on Patient’s Bill of Rights were examined in case and control groups, before, 2 and 4 weeks after an educational intervention program on. Participants were selected from teaching hospitals of Mazandaran University of Medical Sciences, Iran. Data was gathered using the valid and reliable 21-item questionnaire in a 3-point Likert scale during a 5-month period from October 2013 to March 2014. For data analysis, descriptive statistical methods, paired t-test, and repeated measure analysis of variance at significant level P<0.05 were used.

**Results:**

Participants’ mean age and work experience were found 37.1±5.71 years and 11.76±5.99 years respectively. Mean scores of nurses’ awareness and practice before intervention were 15.12±2.19 and 9.13±2.36, accordingly. Repeated measure analysis of variance test showed a significant difference in awareness and practice of nurses before and after intervention (P<0.001).

**Conclusion:**

Enhancing nurses’ awareness on Patient’s Bill of Rights through revision of educational curriculum in nursing schools, together with considering appropriate relevant content in continuous education programs in health systems can lead to improved quality of nursing care services.

## 1. Introduction

Emphasis on human rights in health care system and in particular maintaining patient dignity as a human being becomes important when it is realized that special circumstances of the patient easily expose him to violations and weaknesses of health care system (Soodabeh Joolaee, Nikbakht-Nasrabadi, Parsa-Yekta, Tschudin, & Mansouri, 2006; Woogara, 2005). Patients, as one of society’s vulnerable groups in physical, psychosocial and economical terms are at risk, and this provides the reason for special attention of the international community to the concept of patient’s rights (Woogara, 2005).

Patients have the right to care, treatment, and services that safeguards their personal dignity and respect their cultural, psychosocial and spiritual values. These values often affect the patient’s treatment needs and preferences. By understanding and respecting patients and their values, providers can help meet the patients’ needs for treatment and services and protect the patients. Also defending patient’s rights is essential to maintain his dignity and respect, and if observed, a good relationship can be established between physicians, nurses and patients, and the quality of patient care will improve (Easley & Allen, 2007). In the past, it was imagined that only health care providers were aware of people’s health and well-being and were authorized to decide patients’ destiny. However, today, it is essential to respect basic rights of patients as human beings (Kuzu, Ergin, & Zencir, 2006).

To ensure observing these rights, healthcare systems in different countries have developed guidelines as Patient’s Bill of Rights and have notified it to all executive levels. Accordingly, health care centers are obliged to provide the patient with this Bill at admission, so that clients know their rights and take action to restore them when necessary (Hajavi, Tabibi, & Sarbaz Zeinabad, 2005). Patient’s Bill of Rights states that every patient, regardless of age, gender, race and other differences, has the right to knowledge, respect, confidentiality, privacy, proper care and treatment as well as protection and objection. In this way, health care providers including nurses have the duty to observe these rights. For patients’ rights to be observed, first patients and health care providers should be aware of these rights. Physicians’ and nurses’ lack of awareness leads to their inability to recognize patients’ legal and ethical issues, and reduces the quality of services provided (Alghanim, 2012; Easley & Allen, 2007; Nasiriani, Farnia, & Nasiriani, 2007).

In Iran, Patient’s Bill of Rights was notified for the first time in 2002 by the Health Deputy of the Ministry of Health. Considering the need for development of a comprehensive text on patient’s rights, the bill was revised in 2009 with a holistic view and with the aim to clearly explain rights of recipients of health services, and observing ethical standards in medical area. The bill was developed and notified on five main axes, including: the right to receive appropriate services, the right to receive adequate and appropriate information, the right to freely choose and decide on health services, the right to respect for privacy and confidentiality, and the right to access to efficient complaint system (Hajavi et al., 2005; Nasiriani et al., 2007).

Conducted studies indicate that the most patients are not aware of their rights and nurses’ awareness and practice of patient’s rights in various hospitals is in moderate level and varies from 30% to 60% (Ducinskiene, Vladickiene, Kalediene, & Haapala, 2006; Heidari, Ahmadpour, & GharehBoughlou, 2013; Iltanen, Leino Kilpi, Puukka, & Suhonen, 2012; Kumar, Mehta, & Kalra, 2011; Mohammadnejad, Ehsani, Beigjani, Aboutalebi, & Kalantarzadeh, 2012; Nejad, Begjani, Abotalebi, Salari, & Ehsani, 2011). It has been shown that educational interventions for health care providers can lead to changes of attitude and practice in them, resulting in providing better services to patients. Furthermore, providing education-based interventions for patients and health care providers leads to improved and enhanced observation of Patient’s Bill of Rights, and improved quality of services and patient satisfaction (Eteraf Oskouei, Sadegh Tabrizi, Gharibi, & Asghari Jafarabadi, 2013; Parker, 2013; Saito et al., 2011).

Since health care providers, including nurses will not be able to provide good quality of care without awareness and training about concept and content of patient’s rights, and in attention to very limited number of studies on nurses’ education about Patient’s Bill of Rights, the present study was conducted with the aim to determine the effect of education on level of self-reported awareness and practice of nurses in observing Bill of Rights.

## 2. Methods

### 2.1 Setting and Data Collection

In this quasi-experimental study, 90 nurses employed at 2 teaching hospitals [BU-Ali and Imam Hospitals] affiliated to Mazandaran University of Medical Sciences, Sari, north of Iran, entered the study during a 5-month period from October 2013 to March 2014. Nurse with minimum 2 years of clinical work history and no participation in Patient’s Bill of Rights educational workshops were included. To avoid the effect of education of the case group nurses on the subjects in the control group, one of the centers [Imam] was designated intervention center and the other control. At each center, 45 eligible nurses that were willing to participate were selected according to table of random numbers. At the outset, using relevant questionnaires, a pretest was carried out for both groups to determine nurses’ level of awareness and practice of Patient’s Bill of Rights. Then, in-person education was scheduled for the case group in groups of 8-12 people, with Q & A using a PowerPoint^®^ presentation, and distributing educational pamphlets containing Patient’s Bill of Rights. There were 2 sessions for each group, each lasting 50 minutes. The control group received no intervention. By the end of the second and 4th weeks following intervention, awareness and practice of Patient’s Bill of Rights were reassessed in both groups. The meaning of knowledge and practice in this study was the mean score of

### 2.2 Instrument

Data collection instrument consisted of a self-administered questionnaire comprising 3 parts. The first part was concerned with demographic characteristics of participants; the second part consisted of 21 items to assess nurses’ awareness of patient’s rights. They were included 4 items on the right to knowledge, 3 items on the right to choose and decide, 5 items on the right to confidentiality and privacy, 3 items on the right to respect, 3 items on the right to proper care and treatment, 1 items on the right to complain, and 2 on the right to refuse. Every item assessed nurses’ awareness and scored either 0(wrong answer based on Patient’s Bill of Rights) or 1 (correct answer) mark. The sum total of marks came to minimum of 0 and maximum of 21 which demonstrated the level of knowledge of participants in this project. In the third part, in terms of nurses’ practice on Patient’s Bill of Rights, some items was prepared to assess level of observing patient’s rights from nurses’ perspective, which in terms of frequency and type of situations and overall score was similar to the awareness part. In each item, following stating the situation, a number of options had been proposed, and the most appropriate one, if chosen, scored 1 mark, and otherwise zero. The validity of the questionnaire had been examined in an Iranian study using content validity (Nasiriani et al., 2007) (Waltz and Basel Index found 96% in awareness area and 85% in practice). For the reliability of questionnaire, test re-test method was used, and questionnaire was completed after 2 weeks by 15 nurses. In this way, the second part’s consistency was r=0.95 for awareness of patient’s rights, and r=0.90 for the third part of questionnaire relating to practice.

### 2.3 Data Analysis

After data collection, they were analyzed with SPSS-16 (SPSS Inc., Chicago, IL, USA) using descriptive statistics, chi-square, correlation coefficient, paired t-test, and repeated measure analysis of variance.

## 3. Results

Participants’ mean age and work experience were found 37.1±5.71 years and 11.76±5.99 years respectively. Also, mean scores of awareness and practice of nurses before intervention were 15.12±2.19 and 9.13±2.36, respectively. Table 1 presents characteristics of both group participants. Based on the findings, there was a significant difference between mean awareness scores of the case and the control group 2 weeks after intervention (t=8.28, P<0.001), and 4 weeks after intervention (t=10.24, P<0.001). There was also a significant difference in mean practice scores of the case and the control groups 2 weeks after intervention (t=3.33, P<0.001) and 4 weeks after intervention (t=2.62, P<0.010) (Table 2).

**Table 1:** Socio-demographic characteristics of the participants by case and control group

Characteristics	Case N=50	Control N=43	Sig.	Total N=93
Age*	37.40 ±5.29	36.74±6.21	N.S	37.10±5.71

Sex**				
Female	39(84.7)	42(89.3)	N.S	82(88.2)
Male	7(15.3)	5(10.7)		11(11.8)

Employment record*	12.50±5.62	10.91±6.35	N.S	11.76±5.99

Knowledge score*	15.26±2.26	14.97±2.12	N.S	15.12±2.19

Practice score*	8.82±2.43	9.51±2.26	N.S	9.13±2.36

* Mean± SD;

** Frequency (percent).

**Table 2:** Mean score of knowledge and practice before and after intervention by case and control group

		Groups			

		Case (n=50) Mean±SD	Control (n=43) Mean±SD	t	*P*
Before intervention	Knowledge	15.26±2.26	14.97±2.12	.622	.535

	practice	8.82±2.43	9.51±2.26	1.421	.159

2 weeks after intervention	Knowledge	17.82±1.45	14.88±1.89	8.289	<.001

	practice	10.84±2.16	9.32±2.01	3.330	.001

4 weeks after intervention	Knowledge	18.60±1.47	14.81±2.01	10.245	<.001

	practice	11.30±2.45	9.95±2.46	2.627	.010

Considering that assessment is an ongoing process, and in this study reassessment was performed 2 and 4 weeks after intervention, repeated measure analysis of variance and Bonferroni tests were used to analyze and compare mean scores of awareness and practice of nurses about Patient’s Bill of Rights. The results showed a significant relationship between nurses’ (in case group) awareness scores before and 2 weeks after intervention (P<0.001), before and 4 weeks after intervention (P<0.001) and 2 and 4 weeks after intervention (P=0.001) (table 3). According to findings demonstrated in table 4, there was a significant relationship between these nurses’ practice scores before and 2 weeks after intervention (P<0.001), and before and 4 weeks after intervention (P<0.001). However, no relationship was found in practice scores of this group of nurses, 2 and 4 weeks after intervention (P=0.891).

**Table 3:** Repeated Measurement of knowledge score in case group

(I) Knowledge	(J) Knowledge	Mean Difference (I-J)	Std. Error	Sig.^a^	95% CI^b^
1*	2**	2.56	.29	<.001	3.26-1.83
	3***	3.34	.32	<.001	4.15-2.52

2	1	2.56	.29	<.001	1.83-3.28
	3	.78	.19	.001	1.27-.28

3	1	3.34	.32	<.001	2.52-4.15
	2	.78	.19	.001	.28-1.27

* Knowledge score before intervention;

** Knowledge score 2 weeks after intervention;

*** Knowledge score 4 weeks after intervention;

a. Adjustment for multiple comparisons Bonferroni;

b. Confidence Interval.

**Table 4:** Repeated Measurement of practice scores in case group

(I) Practice	(J) Practice	Mean Difference (I-J)	Std. Error	Sig.^a^	95% CI^b^
1*	2**	1.94	.36	<.001	2.84-1.03
	3***	2.40	.46	<.001	3.54-1.25

2	1	1.94	.36	<.001	1.03-2.84
	3	.46	.43	.89	1.54-.62

3	1	2.40	.46	<.001	1.25-3.54
	2	.46	.43	.891	.62-1.54

* Practice score before intervention;

** Practice score 2 weeks after intervention;

*** Practice score 4 weeks after intervention;

a. Adjustment for multiple comparisons Bonferroni;

b. Confidence Interval.

## 4. Discussion

Receiving medical services combined with reverence and respect is natural and legal right of patients presenting to health care centers which stated in Patient’s Bill of Rights (Easley & Allen, 2007; Woogara, 2005). In some cases, such rights may not be observed, and patient’s rights might be violated. Research and statistics indicate, despite posting the statement of Patient’s Bill of Rights in hospitals, level of observing this Bill is poor to moderate (Ducinskiene et al., 2006; Hajavi et al., 2005). The present study is among few studies conducted with intervention-based approach to enhance awareness and practice of nurses in observing patients’ rights.

The present study, in line with other similar studies shows that before intervention, nurses had moderate level of awareness and practice in observing patients’ rights (Alghanim, 2012; Hakan Özdemir et al., 2009; Heidari et al., 2013; Iltanen et al., 2012). It seems poor awareness and especially poor practice of nurses are rooted from factors such as heavy workload, insufficient time allocated to patients, socioeconomic pressures, and poor awareness of patients as well as health care providers about provisions of Patient’s Bill of Rights (Joolaee, Tschudin, Nikbakht Nasrabadi, & Parsa Yekta, 2008; Kumari, Kumari, & Bishnoi, 2013). In these circumstances, traditional approach, in which health care providers decide for patients, may be more agreeable, which is in conflict with principles of patient’s rights, including the right to choose and decide. Conversely, the presence of patient’s family member, accountability of the health care system, people’s awareness of their rights, and management’s accountability can facilitate observing patient’s rights (S Joolaee et al., 2008). It seems, despite the number of years since issuing Patient’s Bill of Rights, its implementation process has not properly been established, so that, in addition to recipients of health services, nurses are not aware of its contents, and do not properly practice in making decisions in patients’ ethical and legal situations. Accordingly, it is recommended that some of the challenges be resolved through review of how the bill is implemented by policy-makers in health, along with public information. In this way, emphasis on the role of mass media, and attention to obstacles and facilitators in observing Patient’s Bill of Rights is advisable.

Scoring higher score in awareness and practice by nurses that received educational intervention compared to the control group is similar to results of studies that show educational programs can change practice of nurses and ultimately lead to improved quality of care services (Collins, 2013; Parker, 2013; Saito et al., 2011; Sarna et al., 2014; Self, Baldwin Jr, & Olivarez, 1993). Since in Iran’s education system, there is no independent subject titled nursing ethics and observing patient’s rights in the nursing students’ curriculum, it is recommended that the curriculum be revised, and in-service educational courses be considered for nurses. In this respect, it has been shown that the need for education of observing professional ethics and regulations is among the needs of health care providers in continuous educational programs (Abedian, Yazdani Charati, Samadaee, & Shahhosseini, 2014).

In conclusion, implementation of education-based interventions for nurses will lead to improvement in level of awareness and practice in observing Patient’s Bill of Rights. Thus, it is recommended to help improve quality of nursing care services by revising educational curriculum of nursing students, and considering relevant appropriate content in in-service educational programs and enhancing nurses’ awareness of Patient’s Bill of Rights.

Among study limitations was education of nurses through lecture style method. It is recommended that in future studies, similar projects be designed to real nursing workplace environment based on active learning methods like role-play, and simulation to help depth and extent of education. Other limitations included assessment of participating nurses’ practice of observing patient’s rights based on self-reports, which may be different from their assessment in real situation. Considering that the present study is among the few studies with the aim to determine the effect of educational program on awareness and practice of Iranian nurses, it seems, its results as a pilot study can provide the context for future studies in this area.
